# Understanding Perceived Age-Based Judgement as a Precursor to Age-Based Stereotype Threat in Everyday Settings

**DOI:** 10.3389/fpsyg.2021.640567

**Published:** 2021-06-14

**Authors:** Ruth A. Lamont, Hannah J. Swift, Lisbeth Drury

**Affiliations:** ^1^College of Medicine and Health, University of Exeter, Exeter, United Kingdom; ^2^School of Psychology, University of Kent, Canterbury, United Kingdom; ^3^Department of Organizational Psychology, Birkbeck, University of London, London, United Kingdom

**Keywords:** age stereotyping, stereotype threat, threat-based concerns, COVID-19, young, middle age, older adult

## Abstract

Test conditions eliciting negative stereotypes of aging among older adults can prompt age-based stereotype threat (ABST), which results in worse performance on cognitive and memory tests. Much of this research explores ABST as a phenomenon that impacts the performance of older adults. Little is known about the experience of ABST beyond performance settings and how it manifests in everyday contexts across different age groups. Gaps also remain in understanding the wider impacts of ABST, such as effects on task motivation and engagement. The current research addresses this by exploring the contexts in which age-based judgement, a theorized precursor to ABST, occurs across a wide age range of participants. The two studies in this paper present mixed-methods survey data for a total of 282 respondents aged 18–84 years. Study 1 presents a thematic analysis of open-ended responses to identify the stereotypes and settings that underpin perceived age-based judgement. The settings and stereotypes identified are discussed in relation to which contexts lend themselves to adverse ABST effects. Study 2 then asked respondents to rate the extent to which they experience threat-based concern within 12 contexts identified from Study 1. Results indicate differences in threat-based concerns between young, middle-aged and older adults for physical activity, driving, using public transport, using technology, in leadership and relating to the COVID-19 pandemic. The studies provide a foundation for future research to investigate (1) the motivational and behavioural consequences of threat-based concerns for younger adults’ driving and leadership, and in the context of the pandemic; (2) cues to ‘old’ age stereotypes and threat-based concerns among late middle-aged adults within the workplace; (3) the role of broad stereotypes of ‘incompetence’ and being ‘past-it’ on middle-aged and older adults’ engagement with technology and physical activity and (4) potential ABST effects resulting from stereotypes of older people as a burden and a problem in the context of a national crisis. Overall, this research extends our understanding of ABST by identifying further contexts and age groups that could be impacted by a wider range of ABST effects.

## Introduction

More than two decades of experimental research has shown that common stereotypes about aging and older people can influence the behaviours of older people themselves (Meisner, 2011; Westerhof et al., 2014; Lamont et al., 2015). Most of this research focuses on the negative consequences of old age stereotypes that see older adults as less cognitively and physically competent than younger adults (Cuddy et al., 2005; Kite et al., 2005; North and Fiske, 2015). For instance, age-based stereotype threat (ABST) research has shown that older adults who are reminded of these stereotypes tend to perform worse on cognitive performance tests (Lamont et al., 2015). These performance deficits are attributed to emotional, motivational and working memory changes, which arise from a fear of acting in-line with negative stereotype-based expectations (Steele and Aronson, 1995; Schmader et al., 2008; Barber and Mather, 2013). A significant gap in the literature is understanding how this fear of confirming negative age stereotypes manifests outside of the artificial test-based settings in which most ABST research is conducted. Day-to-day, older people will not complete cognitive and physical performance tests as are typical within ABST research, but they may still perceive evaluative scrutiny from others on a wide range of tasks that are relevant to old age stereotypes. The nature and consequence of this perceived scrutiny in everyday scenarios are less well defined. Further, very little ABST research has considered how younger and middle-aged adults experience age stereotypes and if ABST could be problematic for them too (Hehman and Bugental, 2013; von Hippel et al., 2019). In order to address these questions, the first step is to expand our understanding of day-to-day experiences of perceived age-based judgement and threat-based concerns across different age groups and contexts. This should provide important insights into which contexts are potentially problematic for different age groups, because perceived age-based judgement and threat-based concerns are precursory to negative ABST effects (Steele, 2010). Future research can build upon these insights to identify whether these threat-based concerns do disadvantage some age groups over others and what can be done to mitigate these negative effects.

### Age-Based Stereotype Threat

All age groups experience some level of stereotyping across a wide number of domains, but the stereotyping of older people as less competent than the young is most widely recognized and documented cross-culturally (Cuddy et al., 2005; Kite et al., 2005; North and Fiske, 2015; Swift et al., 2019). Consequently, older adults are susceptible to *stereotype threat*, whereby the individual feels at risk of confirming as self-relevant negative group stereotype (Steele and Aronson, 1995). Steele (2010) described stereotype threat as a threat ‘in the air’, suggesting that people are aware of their social identities and the societal stereotypes that are tied to them, but certain settings or cues will make them more salient. For example, in the workplace, the salience of old age stereotypes could vary depending on the workplace age demographic, evaluative pressures (such as workplace assessments or training) and/or the stereotype relevance of a given work context (such as using technology or creative thinking in which older adults are negatively stereotyped). Greater stereotype salience leads to increased feelings of scrutiny, which in turn increases susceptibility to detrimental ABST effects (Steele and Aronson, 1995).

Thus far, the presence of ABST has been predominantly evidenced through the experimental manipulation of stereotype salience (e.g. highlighting age, age comparison or age stereotypes through the introduction) and demonstration of the resulting ‘stereotype threat effects’ on test-like performance (Lamont et al., 2015). Performance decrements on cognitive tests are most commonly attributed to performance inhibiting reactions to age stereotypes, such as changes in working memory and motivational focus (Steele and Aronson, 1995; Schmader et al., 2008; Barber and Mather, 2013). The most up-to-date meta-analysis of this research area shows support for ABST effects on the memory and wider cognitive performance of older adults (Lamont et al., 2015), and some research has investigated how ABST effects physical outcomes, such as walking and grip strength (Lamont et al., 2015; Chiviacowsky et al., 2018; Marquet et al., 2018; Barber et al., 2020). Collectively, ABST research has been vital in demonstrating the detrimental effects of negative age stereotypes for older people and has evidenced the need to reframe and challenge representations of age (Swift et al., 2017). However, it is largely restricted to the artificial manipulation of stereotype salience among older people and the measurement of performance on ‘tests’ as indicative of the experience of ABST. This neglects the wider impact that the experience of ABST, as an identity-related threat, may have in naturalistic everyday contexts, on outcomes beyond ‘performance’ and among younger age groups.

### Examining Age-Based Judgement in Everyday Contexts

Although some people will need to complete cognitive or physical tests as part of workplace or medical assessment (e.g. pre-employment testing, memory, balance or strength tests), this is infrequent. Conversely, informal evaluative contexts, where individuals perform a stereotype-relevant task in the presence of others, are much more likely, for example, when playing sports, using technology, in the workplace, or when driving. Research has shown that the manipulation of ABST has negative consequences for simulated driving performance (Joanisse et al., 2013; Lambert et al., 2016), but not learning outcomes among older people (Fritzsche et al., 2009). Beyond ‘performance’ outcomes, a documented consequence of stereotype threat is described as ‘disidentification’ or ‘disengagement’, characterized by lower intentions to engage in a stereotyped task/domain (Davies et al., 2005). Indicative of disidentification, a growing body of research has shown that ‘older’ or ‘mature’ workers (usually categorized as aged 45/50+ and mean age of mid-50s) who perceive more negative age-based stereotyping in the workplace are also more likely to hold negative work attitudes and show greater disengagement with their work (Kulik et al., 2016; Oliveira and Cabral-Cardoso, 2018; Manzi et al., 2019; von Hippel et al., 2019). In addition, one study found that making age stereotypes salient reduced older people’s subjective assessment of their own hearing ability (Barber and Lee, 2016). Therefore, in addition to affecting performance-based tasks, ABST effects may manifest in informal evaluative contexts and also as maladaptive coping strategies, including reduced engagement and depleted self-evaluation within stereotyped domains. It is encouraging that research explores the wider ramifications of ABST, but these studies remain focused on the consequences of experienced threat for older people/workers. There is still little understanding of how ABST is experienced across a wider age range, or in relation to other age stereotypes.

Only two published studies have tested ABST among younger age groups. The first found a ‘stereotype challenge’ effect among younger adults (aged 17–22 years old), whereby they performed better on an intelligence test when told success was dependent on wisdom and life experience (positive old age stereotypes as a manipulation of threat), if they also felt in control (Hehman and Bugental, 2013). In a survey of everyday perceptions of stereotype threat, a second study found that although participants across age groups (aged 18–66 years old) reported ABST events (experiences of being negatively evaluated based on age) in the workplace, this was only linked to disengagement at work among older workers aged 50+ (von Hippel et al., 2019). These graver consequences of ABST for older versus younger adults are generally accounted for by the impermanence of being ‘young’ and so an ability to ‘resist’ or ‘challenge’ age stereotypes and the threat they pose (Hehman and Bugental, 2013). However, research has not sought to understand younger or middle-aged groups’ perceptions and experience of age stereotyping and the stereotypes they might be most threatened by.

A precursor to stereotype threat effects is the perception of identity-based evaluative scrutiny from others (Steele, 2010), which in relation to age, we call *perceived age-based judgement*. To fully understand the implications of ABST and its relevance to the day-to-day lives of people, we argue that there is a need to understand the broader contexts and domains in which age-based judgement is perceived, and define its experience across the life-course. Prior research has established that ABST can disadvantage older adults on formal performance tests, therefore, the studies within this paper add to the literature by exploring experiences of age-based judgement in naturalistic everyday contexts, on outcomes beyond ‘performance’ and among younger age groups. Then, we explore whether people feel threatened by these experiences to broaden the scope and relevance of ABST research. By developing theory in this way, the current studies provide a clearer focus for future ABST research so that it may realize the lived experience of ABST across the life-course.

Study 1 provides a qualitative exploration of the nature of perceived age-based judgements, and the stereotypes people believe are applied to their age group and in what contexts. The previous research is used to infer which areas of age-based judgement are likely to have negative motivational and behavioural consequences. The findings from Study 1 are then used to inform Study 2, which provides a quantitative assessment of how everyday contexts in which age-based judgement is perceived, as identified in Study 1, are linked to the threat-based concerns of different age groups. A deeper understanding of the experience of age-based judgement and threat-based concerns will form the basis for future experimental ABST research with greater relevance to everyday experience.

## Study 1

To explore *perceived age-based judgement*, study 1 asks respondents to reflect on the stereotypes applied to their age group and the contexts these are applied in. Importantly, to reflect the subjective nature of age group categorization (Abrams et al., 2009), the study explores respondents age in relation to their own perceived age grouping. These findings are then interpreted and informed by the previous ABST research and wider theories of stereotype threat. According to stereotype threat theory, if these stereotypes devalue the individual it is possible for them to be threatening, particularly if they are negative about the competencies of the group. Alternatively, stereotypes that place positive expectations on an individual are ‘identity-safe’ and not considered triggers to stereotype threat (Purdie-Vaughns et al., 2008). Further, when a stereotype relates to a context in which cognitive resources may be important for outcomes, negative ABST effects on these outcomes (such as underperformance) will be more likely (Schmader et al., 2008). Finally, the earlier discussed research findings for younger adults experiencing stereotype threat would indicate that if there is potential for change (e.g. elimination of negative stereotypes *via* aging), negative stereotypes of that age group may not lead to aversive outcomes (Hehman and Bugental, 2013; von Hippel et al., 2019).

### Method

#### Participants and Procedure

We employed a quasi-experimental design to compare the responses of different age groups on open-ended measures of perceived age-based judgement. The measures analysed were taken from a larger survey on perceived judgement from others (University of Kent, School of Psychology ethics reference: 20122324). Data were collected for 118 respondents, but thirteen were removed due to missing data on key variables pertaining to this study, predominantly participant age. Over half of respondents were female (*n* = 66; 62.9%), almost all were ‘white’ (97.1%) and ages ranged from 18 to 83 years old (*M*_age_ = 51.12; *SD* = 20.37). Participants were recruited online (*n* = 36; 34.3%) and using paper questionnaires sent by post to community groups in the South East England (*n* = 69; 65.7%). Both formats were completed independently of the researchers.

#### Measures

To start, respondents were asked how they would categorize their age group (subjective age group): *‘What would you call the age group that you feel part of?’ e.g. a 12-year-old may say they are a ‘child’ or ‘teenager’*. Respondents gave free responses to this question. Following this, a number of items were used to look at people’s perceptions of age-based judgement. Respondents were asked about the *stereotypes* they think others hold about their age group: *‘What do you think are the most common stereotypes (popular/common beliefs or expectations) that are held by others about your age group?’*. Space for up to five free responses was given. Respondents were then asked about the *settings* in which they think age-based judgement occurs *‘In what area/s of your life (e.g. this may be on a specific task/activity/action or more general) do you feel others judge you differently based on your age?’*. The question allowed for two open-ended responses. The questions were deliberately open to allow participants to think of both positive and negative experiences and were devised by the authors for the purpose of this research.

#### Analyses

Responses to subjective age group questioning were ordered based on participant age, and thematic shifts in descriptions of age groupings were used as indication of where these subjective age groups start and end. Qualitative data from age-based judgement questions on stereotypes and setting were analysed using inductive thematic analysis, whereby patterns (themes) in the data were identified without a preexisting coding framework, allowing for the data to be examined in all its richness (Braun and Clarke, 2006). Open-ended responses were largely one-word answers or a short phrase, allowing words to be easily categorized looking for repeated patterns in meaning, or common views expressed by respondents. Two researchers separately coded all participant responses and discrepancies in naming of themes and the breadth of themes were resolved through discussion. A third coder then used these themes to deductively code all participant responses once more. High inter-rater reliability between the third coder and the themes assigned by the primary coder for both stereotypes (Cohen’s Kappa or *κ*; *κ* = 0.86) and settings (Cohen’s Kappa or *κ*; *κ* = 0.92) gives confidence in the use of these themes (Cohen, 1960).

### Results

#### Age Groups

Two 18-year-olds described their age group as ‘teenager’, while the majority of those aged 19 to 31 described themselves as ‘young adult’, ‘young professional’, ‘student’ or ‘twenty something’. Consistent with Abrams et al. (2009), respondents stopped describing themselves as ‘young’ and started to use the description of ‘adult’ or ‘early/middle/late thirties’ around the age of 32. The term ‘middle-aged’ then began to be used in the late 30s, with this being the predominant descriptive until the age of 59. Consistent with Sweiry and Willitts (2012), those in their early 60s started to use descriptive terms related to the next stage of life, but often caveated, for example, ‘young/active/early retired’. Various terms referring to old age were then used among respondents from the mid-60s, including ‘retired’, ‘older person’ and ‘pensioner’. The terms ‘elderly’, ‘senior citizen’ and ‘OAP’ were most common among those over 70. Age groups were therefore created, aligning with the transitions of these common descriptions; those aged 18 to 31 are described as ‘young adults’, those aged 32 to 59 as ‘middle-aged adults’, those aged 60–69 as ‘early older-aged adults’ and those aged 70 and above as ‘late older-aged adults’ (Table 1).

**Table 1:** Study 1: Demographic information split by subjective age groupings.

Age group	Age range	*N*	% completed online	% female	*M* age (*SD*)
Young	18–31	27	70.4%	77.8%	23.52 (3.83)
Middle-aged	32–59	30	76.7%	58.6%	45.67 (8.38)
Early older-aged	60–69	25	72%	60%	64.80 (3.22)
Late older-aged	70+	23	39.1%	56.5%	75.78 (4)

#### Perceived Age-Based Judgement

**Figure 1:**
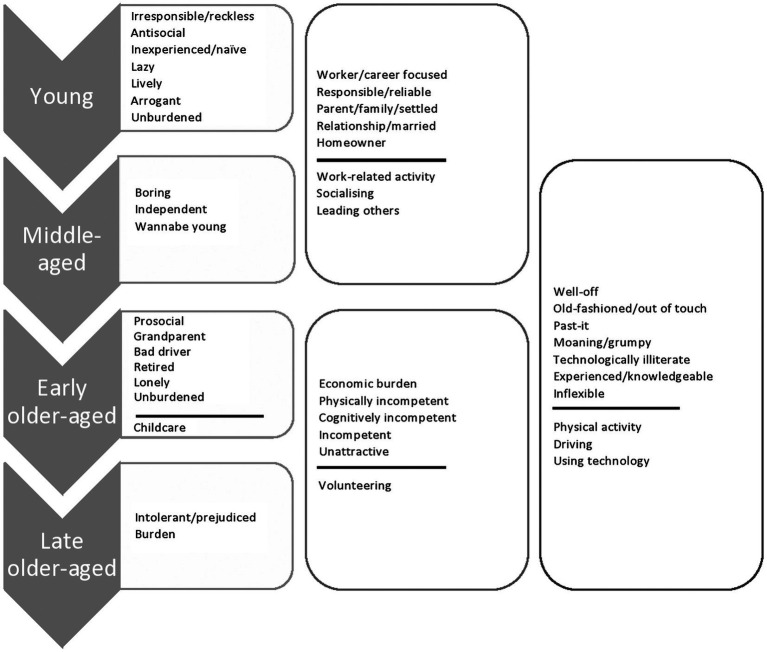
Study 1: Age-based judgement themes.

The themes identified from open-ended responses are detailed in Figure 1 (stereotypes above the line and settings below).

##### Young and reckless…

Younger adults overwhelmingly perceived that they were stereotyped as demonstrating ‘*irresponsible*’ and ‘*reckless*’ behaviours (‘*party people*’, ‘*binge drinkers*’, ‘*poor financial control*’ and ‘*promiscuous*’). In a similar vein, younger adults perceived that their age group was stereotyped negatively as ‘*antisocial*’ towards others (‘*non-respectful of adults*’, ‘*intimidating*’ and ‘*rude*’), ‘*inexperienced*’/‘*naïve*’, ‘*arrogant*’ and ‘*lazy*’. For younger adults (and others of working age), age-based judgement was perceived in the workplace and more specifically in the context of leadership. Stereotype threat theory would suggest that perceiving stereotypes of irresponsibility, laziness and inexperience may pose a threat to younger adults, leading to underperformance in work and leadership contexts (Steele, 2010). However, previous research shows that perceived age-based judgement in the workplace reported by younger adults is less likely to lead to negative ABST effects and instead encourages a ‘challenge’ response, purportedly due to the anticipated transition to middle-age (von Hippel et al., 2019). This is supported by the coupling of negative stereotypes of ‘youth’ behaviour with stereotypes of being ‘*lively*’ (‘*energetic*’, ‘*loud*’, ‘*fun loving*’ and ‘*sociable*’) and a number of more neutral prescriptive stereotypes that cross-over with the middle-aged and relate to life circumstance.

##### …closely followed by middle-aged and settled…

Being settled in a relationship/married, with children, a house, and work or a career (‘*career focused*’, ‘*hard workers*’, ‘*should be married*’, ‘*you should have a partner*’, ‘*settled down*’, ‘*you should have kids*’ and ‘*own a house*’), and being ‘*responsible*’/‘*reliable*’ and independent (‘*maturing*’, ‘*sensible*’, ‘*self-sufficient*’ and ‘*independence*’) were provided by many of the young respondents and gained prominence among the middle-aged. These stereotypes are less negative and more descriptive, outlining standards which the young to middle-aged think they are expected to achieve. This suggests that middle-aged adults may feel pressures to achieve these components of adult life; however, these stereotypes do not attribute negative ability to young and middle-aged adults and so according to stereotype threat theory are unlikely to produce ABST effects of underperformance or disengagement (Steele, 2010).

##### …and before you know it, you are past-it

Whereas ABST research has typically examined the threat of old-age/aging stereotypes among those aged 65+ (Lamont et al., 2015), participant responses show that concerns around these old-age stereotypes start much earlier. As early as middle-age, participants believed that they may be stereotyped as boring and harking back to their younger days (‘*trying to be younger than we are*’ and ‘*desperate for youth’*). They believed they were stereotyped as ‘*old-fashioned*’ and ‘*out-of-touch*’ (‘*not in touch with the modern world*’, ‘*lives in the past*’ and ‘*conservative*’) and ‘*past-it*’ (‘*getting a bit past their best*’ and ‘*redundant*’). Other negative age stereotypes began to appear, such as being ‘*moaning*’/‘*grumpy*’ (‘*always complaining*’ and ‘*grumbly*’), unable to use modern technology (‘*cannot cope with new technology*’ and ‘*technologically illiterate*’) and being ‘*inflexible*’ (‘*set in ways*’ and ‘*against change*’). Perceptions of these negative age stereotypes peaked in early older-age and continued into late older-age. Given that the middle-aged also perceived the workplace, and using technology as key settings for age-based judgement, these negative stereotypes of being past-it, not modern and technologically illiterate may lead to ABST effects and reluctance (disidentification) when engaging with technology, new tasks and at work (Davies et al., 2005; von Hippel et al., 2019). Unlike younger adults, these stereotypes do not go away with age and so may present a threat to middle-aged adults (von Hippel et al., 2019). ABST has yet to be examined among this group.

##### Declining competence with age

Early and late older-aged respondents strongly believed they are stereotyped as physically (‘*slower*’, ‘*deaf*’, ‘*infirm*’ and ‘*weak*’), cognitively (‘*forgetful*’, ‘*senile*’, ‘*decreasing mental capacity*’ and ‘*slow and not very bright*’) and more broadly incompetent (‘*old duffers*’, ‘*dithery*’ and ‘*slow/bad drivers*’). This ties in with the bulk of ABST research to date which has examined how the stereotyping of older adults as incompetent affects their performance on memory/cognitive tasks, but also some physical performance tasks (Lamont et al., 2015). However, beyond these formal test-based settings, responses highlight that early and late older-aged adults (and some middle-aged) feel subject to age-based judgement while at work, using technology (as discussed), driving and doing physical activity, while volunteering and providing childcare. A wide number of other everyday contexts (e.g. DIY, healthcare, public speaking and doing various hobbies) were less frequently, but still importantly listed as places for potential age-based judgement among older respondents. These broadly perceived stereotypes of incompetence, as well as the many informal performance contexts in which people are conscious of these, present a number of un-examined everyday settings in which ABST may be problematic (Lamont et al., 2015). Although some middle-aged to late older-aged adults did acknowledge the positive stereotyping of their age groups’ as more ‘*experienced*’/‘*knowledgeable*’ (‘*wise*’ and ‘*someone who can pass-on skills*’), it is unclear whether these would be enough to buffer against the rife perceived judgements of incompetence.

##### Doddering but dear

Alongside the surplus of negative competency stereotypes, those in early old-age often believed they were stereotyped as prosocial (‘*trustworthy*’, ‘*source of volunteers*’, ‘*supportive*’ and ‘*friendly*’). Unlike stereotypes of being experienced and knowledgeable, these pro-social stereotypes do not directly link to ‘ability’ and so are unlikely to be protective of ABST effects (Steele, 2010). The *Stereotype Content Model* (Cuddy et al., 2005) which aligns with these findings suggests that as we get older we are more likely to be stereotyped as ‘doddering but dear’, incompetent but friendly. This combination of stereotypes has been linked to the pitying of older people and benevolent ageism towards them (Cuddy et al., 2005, 2008), which is consistent with some of the low-status or belittling stereotypes this group perceive as ‘*grandparents*’ (‘*free childcare*’ and ‘*live for grandchildren*’), ‘*unattractive*’ (‘*wrinklies*’ and ‘*not so attractive*’), ‘*lonely*’ and arrogant in the sense that they ‘*think they know best*’. The addition of positive prosocial stereotypes is therefore unlikely to add to the status or esteem felt by older people in evaluative contexts.

##### A burden to others

The most notable stereotype salient among late older-aged adults was that of being an economic burden. Late older-aged adults believed they were seen as ‘*bed-blockers*’ in health and social care, ‘*economically unproductive*’, ‘*hogging property wealth*’ and receiving ‘*generous pensions*’. This stereotype of older adults as an economic burden is linked to both stereotypes of incompetence (and therefore reduced productivity), but also stereotypes of older adults as well-off and over-privileged (Cuddy et al., 2005). Our older respondents believed they were stereotyped as ‘*well-off*’ (‘*plenty of money*’, ‘*better off*’, ‘*affluent*’ and ‘*had a very easy life economically*’) and ‘*unburdened*’ (‘*easy life*’, ‘*time on their hands*’ and ‘*leisurely*’). Older adults were also the main age group to report stereotypes of intolerance/prejudice, often characterized as an intolerance or prejudice towards the young (‘*intolerant of younger people*’, ‘*prejudiced*’ and ‘*we are less tolerant*’). Research has not yet examined how stereotypes relating to older people as a burden or prejudiced might relate to ABST. It is possible that both may encourage disengagement that could negatively impact the health and wellbeing of older people, for example, disengagement with younger people for fear of awkward interaction or disengagement with health and social care for fear of being a ‘burden’ (Davies et al., 2005; von Hippel et al., 2019).

### Conclusion

This study provides the first in-depth analysis of how people perceive age-based judgement across the adult age spectrum. Identified themes from the study were discussed in terms of the likely threat posed to each age group by the age stereotypes/contexts (Purdie-Vaughns et al., 2008), whether this threat is likely to lead to changes in motivation and behaviour. However, to clarify the likelihood of perceived age-based judgement in these domains leading to threat-based concerns, a second study was conducted. Study 2 tests the likelihood of ABST effects within the settings and stereotypes from Study 1 that have been identified (based on the previous theory) as likely to pose a risk to the motivations and behaviours of different age groups.

## Study 2

Study 2 explores *threat-based concerns* in everyday settings (informed by Study 1). The aim is to further define experiences of threat against participant age group and domain. Surprisingly, only a handful of studies have operationalized and measured experiences of threat in ABST studies. Seven out of 22 manuscripts featured in the most comprehensive meta-analysis to date measured self-reported ABST relating to specific performance tests, sometimes termed ‘threat-based concerns’, ‘threat concern’ or ‘perceived stereotype threat’ (Lamont et al., 2015). These measures were included within these studies to confirm the existence of ABST and link it to any resulting performance deficits. Other research has similarly used self-reporting to examine perceived stereotype threat within the workplace (Kulik et al., 2016; Phibbs and Hooker, 2017; Oliveira and Cabral-Cardoso, 2018; Manzi et al., 2019; von Hippel et al., 2019). These studies tend to measure the perceived experience of threat—e.g. items, such as ‘*were you worried that your ability to perform well on the test was affected by your age?*’ and ‘*were you worried that if you performed poorly on the test, the research would attribute your poor performance to your age?*’*—*but do not assess any potential changes in behaviour or motivation based on these threat appraisals. In this study, we extend this measurement of threat-based concerns to enable us to infer the likelihood that a given context will pose a threat, but also present a risk of ABST effects on motivational and behavioural outcomes. In each of the settings explored, participants are asked if they have been aware of their age, aware that others may judge them negatively based on it, whether they have feared confirming negative age stereotypes, and if they have avoided the particular setting because of this.

### Method

#### Design

Using a quasi-experimental design, we tested whether threat-based concerns related to 12 different contexts, varied by respondent’s age and subjective age (to explore if there is corroboration of results).

#### Participants and Procedure

Anyone above the age of 18 was permitted to take part in the survey, and in total, 186 participants were recruited through Prolific Academic in a study titled ‘age awareness in everyday contexts’. The study was approved by the University of Kent, School of Psychology ethics reference: 202016014841796628. Using G*power 3.1, a necessary sample size estimate of 102 was calculated, with 80% power, alpha at 0.5, and assuming a partial eta-squared of 0.09 based on the previous research showing the relationship between age and threat-based concerns (Gaillard et al., 2011). The study was hosted by Qualtrics online survey software. Participants first gave informed consent and then completed the domain-specific threat-based concern questions, followed by demographic information and debrief. Nine participants were removed for failing an attention check. The final sample included 177 participants aged 18 to 82 (*M*_age_ = 45.71; *SD* = 18.36), 54 (30%) identified as male, 122 (69%) as female and a one preferred not to say.

#### Measures

##### Domain-specific threat-based concerns

Participants were asked ‘*When [setting], to what extent in the last year have you…*’, ‘*felt aware of your age*’, ‘*been aware others’ might be judging you negatively because of your age*’, ‘*thought you might confirm a negative stereotype about your age-group*’ and ‘*avoided [setting] because of your age*’. Twelve different settings taken from Study 1 were rated using a 7-point scale (1 = *not at all* to 7 = *very much*). The domains rated were work (‘*working remotely or engaging in paid work*’), physical activity (‘*engaging in physical activity or exercise*’), driving (‘*driving*’), technology (‘*using or learning new technology*’), leisure activity (‘*doing leisure activities or hobbies*’), childcare (‘*looking after children*’), leading others (‘*in a leadership position*’), learning (‘*learning new things*’) and healthcare (‘*engaging in the healthcare system or with healthcare professionals*’). To reflect that participants may not drive, we added public transport (‘*using public transport*’) as a domain. Due to the timelines of the Study 2 data collection during the COVID-19 pandemic, we thought it pertinent to assess also the threat that this situation presents to older people in particular (‘*To what extent has the COVID pandemic made you…*’). As a group, older people have been singled-out as ‘at risk’ and those to be ‘shielded’, which has raised concerns around ageism towards this group during the pandemic (Ayalon, 2020; Morrow-Howell et al., 2020). This last item was not delivered using the same ‘*When you are…*’ prefix as the other items. Cronbach’s alpha indicated very good reliability for all scales >0.78 (see Table 2).

**Table 2:** Study 2: Descriptive statistics and bivariate correlations for age-based stereotype threat domains.

S. No.		*M*	*SD*	Cronbach’s alpha	1	2	3	4	5	6	7	8	9	10	11	12
1	Age	45.71	18.36	−												
2	Gender			−	−0.07											
3	Work	3.15	1.45	0.782	−0.15	0.08										
4	Physical activity	3.19	1.52	0.856	0.36^**^	0.05	0.40^**^									
5	Driving	2.28	1.41	0.848	−0.27^**^	0.16^***^	0.40^**^	0.30^**^								
6	Public transport	1.98	1.23	0.84	−0.05	0.09	0.53^**^	0.42^**^	0.59^**^							
7	Technology	2.63	1.43	0.838	0.18^*^	0.05	0.21^**^	0.40^**^	0.23^**^	0.37^**^						
8	Leisure	2.34	1.31	0.853	−0.08	0.01	0.43^**^	0.49^**^	0.54^**^	0.54^**^	0.51^**^					
9	Children	2.71	1.4	0.83	−0.16^***^	0.11	0.56^**^	0.34^**^	0.55^**^	0.53^**^	0.36^**^	0.46^**^				
10	Leadership	2.89	1.59	0.866	−0.46^**^	0.15	0.40^**^	0.13	0.66^**^	0.45^**^	0.27^**^	0.44^**^	0.45^**^			
11	Learning	2.54	1.35	0.835	0.00	0.07	0.25^**^	0.37^**^	0.34^**^	0.34^**^	0.63^**^	0.51^**^	0.36^**^	0.46^**^		
12	Healthcare	2.8	1.45	0.827	0.03	0.09	0.44^**^	0.45^**^	0.57^**^	0.50^**^	0.37^**^	0.46^**^	0.46^**^	0.48^**^	0.54^**^	
13	COVID	3.42	1.71	0.818	0.01	0.08	0.41^**^	0.31^**^	0.52^**^	0.49^**^	0.28^**^	0.47^**^	0.34^**^	0.50^**^	0.40^**^	0.45^**^

* Correlation is significant at the 0.05 level.

** Correlation is significant at the 0.01 level.

*** Correlation is between 0.05 and 0.07.

*M*, mean; *SD*, standard deviation.

##### Demographic variables

Gender, age, nationality and subjective age (‘*How old do you feel?*’) were measured. Although participant age and subjective age correlated very strongly (*r* = 0.83; *p* < 0.001), we wanted to explore both the effect of chronological and subjective age group on perceived threat in each domain. This is especially important given that subjective age might be a protective factor in experiencing threat. Therefore, both chronological age and subjective age were used to create age group and subjective age group variables. We used age boundaries defined by Study 1 (those aged 18 to 31 are described as ‘young adults’, those aged 32 to 59 as ‘middle-aged adults’, those aged 60 to 69 as ‘early older-aged adults’ and those aged 70 and above as ‘late older-aged adults’), but this resulted in low numbers of participants in the late older-aged group based on chronological age (*n* = 15) and subjective age (*n* = 6). Therefore, we collapsed the last two age groups into an ‘older adults’ age group. This age group categorization is also fairly consistent with perceptions of age groups in the United Kingdom using nationally representative data (Abrams et al., 2009; Sweiry and Willitts, 2012) and resulted in 55 young, 62 middle-aged and 60 older participants. Age group categorization based on subjective age was skewed towards the young age group and resulted in more uneven distribution between age groups: 73 participants in the young age group (feeling 18–31 years old), 74 participants in the middle-aged group (32–59) and 30 participants in the older age group feeling 60 and older.

#### Analyses

IBM SPSS Statistics 26 was used to conduct analyses. Separate univariate ANOVAs were used to examine the associations between age group and subjective age group on age-based stereotype threat reported in each domain.

### Results

Descriptive statistics and bivariate correlations for age-based stereotype threat variables are reported in Table 2. Participant gender did not significantly correlate with feelings of threat in any of the domains and, therefore, was not added as a covariate. Threat-based concerns were highest for the pandemic, followed by work and physical activity domains, which reached the mid-point of the scale. For all other domains, mean scores were lower than the mid-point of the scale, indicating low threat-based concern in general. However, we expect experiences would differ according to participant age group.

#### Chronological Age Group Differences

ANOVAs for all outcomes are shown in Table 3. Analyses reveal significant effects of chronological age group on threat experienced in the domains of physical activity [*F* (2, 171) 10.73, *p* < 0.001, *η*^2^ = 0.11], driving [*F* (2, 145) 12.70, *p* < 0.001, *η*^2^ = 0.15], public transport [*F* (2, 151) 6.23, *p* = 0.003, *η*^2^ = 0.08], leadership [*F* (2, 111) 15.95, *p* < 0.001, *η*^2^ = 0.22] and threat experienced during the pandemic [*F* (2, 174) 6.72, *p* = 0.002, *η*^2^ = 0.07]. Participant age had a marginal effect in the technology domains [*F* (2, 173) 2.56, *p* = 0.080, *η*^2^ = 0.03]. There was no effect of participant age group for the domains of work, leisure, childcare, learning or healthcare.

**Table 3:** Means, standard deviations and univariate ANOVA for perceived age-based stereotype threat by chronological age.

	Chronological age groups						*F*, *p*, *η*^2^
	18–31 years		32–59 years		60 plus		
	*M*	*SD*	*M*	*SD*	*M*	*SD*	
Work	3.49	1.39	2.99	1.37	2.97	1.55	*F* (2, 157) 2.20, *p* = 0.115, *η*^2^ = 0.03
Physical activity	2.46^a^	1.29	3.39^b^	1.44	3.66^b^	1.58	*F* (2, 171) 10.73, *p* < 0.001, *η*^2^ = 0.11
Driving	3.08^a^	1.54	1.81^b^	0.99	2.03^b^	1.36	*F* (2, 145) 12.70, *p* < 0.001, *η*^2^ = 0.15
Public transport	2.27^a^	1.38	1.51^b^	0.73	2.16^a^	1.36	*F* (2, 151) 6.23, *p* = 0.003, *η*^2^ = 0.08
Technology	2.29^a^	1.36	2.67^ab^	1.48	2.88^b^	1.41	*F* (2, 173) 2.56, *p* = 0.080, *η*^2^ = 0.03
Leisure activity	2.56	1.35	2.26	1.31	2.23	1.28	*F* (2, 172) 1.10, *p* = 0.335, *η*^2^ = 0.01
Childcare	3.09	1.53	2.54	1.17	2.50	1.44	*F* (2, 126) 2.42, *p* = 0.093, *η*^2^ = 0.04
Leading others	3.77^a^	1.66	2.32^b^	1.13	2.16^b^	1.32	*F* (2, 111) 15.95, *p* < 0.001, *η*^2^ = 0.22
Learning	2.46	1.25	2.65	1.51	2.52	1.27	*F* (2, 171) 0.30, *p* = 0.740, *η*^2^ = 0.00
Healthcare	2.88	1.64	2.62	1.40	2.91	1.34	*F* (2, 164) 0.69, *p* = 0.503, *η*^2^ = 0.01
Pandemic	3.73^a^	1.86	2.80^b^	1.48	3.78^a^	1.63	*F* (2, 174) 6.72, *p* = 0.002, *η*^2^ = 0.07

Different letters in the same row indicate significant pairwise comparisons *p* < 0.05.

For physical activity, significant pairwise comparisons (*p* < 0.05) reveal that younger participants (*M* = 2.46; *SD* = 1.29) report less threat-based concern compared to older (*M* = 3.66; *SD* = 1.58) and middle-aged (*M* = 3.39; *SD* = 1.44) participants, while middle-aged and older participants do not differ from one another (*p* > 0.05). For driving, younger participants report more threat-based concern (*M* = 3.08; *SD* = 1.54) compared to middle-aged (*M* = 1.81; *SD* = 0.99) and older (*M* = 2.03; *SD* = 1.36) participants, which do not differ from one another. For public transport, threat-based concern was lower for middle-aged participants (*M* = 1.51; *SD* = 0.73) compared to younger (*M* = 2.27; *SD* = 1.38) and older participants (*M* = 2.16; *SD* = 1.36) who do not differ from each other. For leadership, younger participants report greater threat-based concern (*M* = 3.77; *SD* = 1.66) compared to middle-aged (*M* = 2.32; *SD* = 1.13) and older participants (*M* = 2.16; *SD* = 1.32), which do not differ from one another. During the pandemic, younger (*M* = 3.73; *SD* = 1.86) and older participants (*M* = 3.78; *SD* = 1.63) report more COVID-19-induced threat-based concern than middle-aged participants (*M* = 2.80; *SD* = 1.48). For technology, the marginal effect is driven by older participants feeling more threat-based concern (*M* = 2.88; *SD* = 1.41) compared to younger participants (*M* = 2.29; *SD* = 1.36), with middle-aged participants landing in between (*M* = 2.67; *SD* = 1.48).

In sum, younger participants report less threat-based concern than middle-aged and older participants in relation to physical activity, and more threat-based concern than middle-aged and older participants in relation to driving and leadership. Both younger and older participants report more threat-based concern than middle-aged participants in the domains of public transport and in relation to the COVID-19 pandemic, while older participants report more threat-based concern regarding technology than younger, but not middle-aged participants.

#### Subjective Age Group Differences

The analysis with subjective age groups confirmed a similar pattern of results with the exception (Table 4) that the main effect of subjective age group for the workplace domain is marginally significant, while the effect on technology is significant [*F* (2, 173) 5.46, *p* = 0.005, *η*^2^ = 0.06]. For the workplace, the marginal effect is driven by participants that feel younger reporting more threat-based concern (*M* = 3.43; *SD* = 1.40) than middle-aged (*M* = 2.87; *SD* = 1.39) and older participants (*M* = 3.08; *SD* = 1.44), which do not differ from each other. For technology, participants that feel younger report significantly less threat-based concern (*M* = 2.21; *SD* = 1.23) than middle-aged (*M* = 2.89; *SD* = 1.55) and older participants (*M* = 2.97; *SD* = 1.35), which do not differ from one another. In addition, when using subjective age group categorizations, threat-based concern related to the pandemic was more pronounced for participants feeling older, for participants feeling 60 and over (*M* = 4.03; *SD* = 1.55). Therefore, when using subjective age group categorizations, younger participants report marginally more threat-based concern in relation to the workplace, but significantly less threat-based concern in relation to technology.

**Table 4:** Means, standard deviations and univariate ANOVA for perceived age-based stereotype threat by subjective age.

	Subjective age group						*F*, *p*, *η*^2^
	18–31 years		32–59 years		60 plus		
	*M*	*SD*	*M*	*SD*	*M*	*SD*	
Work	3.43^a^	1.40	2.87^b^	1.39	3.08^a^	1.44	*F* (2, 157) 2.64, *p* = 0.074, *η*^2^ = 0.03
Physical activity	2.70^a^	1.27	3.47^b^	1.58	3.75^b^	1.62	*F* (2, 171) 7.53, *p* = 0.001, *η*^2^ = 0.08
Driving	2.73^a^	1.52	1.71^b^	0.93	2.64^a^	1.66	*F* (2, 145) 10.06, *p* < 0.001, *η*^2^ = 0.12
Public transport	2.07^ab^	1.27	1.71^a^	1.05	2.38^b^	1.49	*F* (2, 151) 2.63, *p* = *0*.076, *η*^2^ = 0.03
Technology	2.21^a^	1.23	2.89^b^	1.55	2.97^b^	1.35	*F* (2, 173) 5.46, *p* = 0.005, *η*^2^ = 0.06
Leisure activity	2.42	1.26	2.16	1.26	2.59	1.52	*F* (2, 172) 1.37, *p* = 0.256, *η*^2^ = 0.02
Childcare	2.86	1.42	2.55	1.34	2.71	1.51	*F* (2,126) 0.69, *p* = 0.503, *η*^2^ = 0.01
Leading others	3.46^a^	1.62	2.16^b^	1.23	2.67^ab^	1.58	*F* (2, 111) 9.78, *p* < 0.001, *η*^2^ = 0.15
Learning	2.44	1.18	2.58	1.45	2.70	1.51	*F* (2, 171) 0.42, *p* = 0.656, *η*^2^ = 0.01
Healthcare	2.72	1.53	2.79	2.79	2.99	1.22	*F* (2, 164) 0.36, *p* = 0.696, *η*^2^ = 0.00
Pandemic	3.53^ab^	1.84	3.07^a^	1.57	4.03^b^	1.55	*F* (2, 174) 3.71, *p* = 0.027, *η*^2^ = 0.04

Different letters in the same row indicate significant pairwise comparisons *p* < 0.05.

## Discussion

Previous experimental research has examined the consequences of stereotype threat for older people’s cognitive and physical performance in formal test-based settings (Lamont et al., 2015; Barber et al., 2020). However, outside of the laboratory, our understanding of the everyday contexts that may present an evaluative threat is limited. According to stereotype threat theory, the repercussions or ‘threat effects’ may also reach beyond ‘test performance’ and impact self-evaluations, motivation and engagement and also have the potential to affect more than just ‘older’ adults through age stereotypes relevant to other age groups (Davies et al., 2005; Steele, 2010). To extend our understanding of ABST, the current research sought to qualitatively shed some light on the experience of age-based judgement in everyday contexts among adults of varying age (Study 1) and then quantitatively explore threat-based concerns within these contexts, using an extended measure that takes into account experiences of disidentification (Study 2).

Thematic analysis of open-ended responses in Study 1 showed that most relevant stereotypes for ABST among the young were those of irresponsibility, laziness and inexperience. Alongside this, the work and leadership contexts were named as places that age-based judgement occurred. These negative stereotypes are relevant to being perceived as incompetent within the workplace and in leadership, and therefore have the potential to pose a threat to young people’s identity (Steele, 2010). Adding to this, Study 2 also found that younger participants reported significantly higher threat-based concerns than other age groups in the context of leadership, but also for driving. This is the first research to identify more specific areas of threat-based concern relevant to the young and warrants examination to understand whether these contexts might give rise to negative outcomes for the young or, as previously reported, provoke a challenge response (Garstka et al., 2004; Hehman and Bugental, 2013; von Hippel et al., 2019). The young may indeed overcome threatening stereotypes even in the contexts in which they are most salient to them, or it may be the case that previous research inclusive of the young did not highlight stereotypes or specific contexts relevant enough to them to provoke a threat response when using positive stereotypes of the old instead (Hehman and Bugental, 2013; von Hippel et al., 2019).

To date, no ABST research has examined the impact of negative age stereotypes relating to middle-aged adults, often seeing them as the privileged, high-status age group (Garstka et al., 2004; Abrams et al., 2011). Thus, adults of all ages were included in this research. Adding to the knowledge base on age stereotypes, Study 1 revealed that middle-aged adults perceived predominantly prescriptive stereotypes, reflecting expectations relating to work, responsibility and relationships. However, this age group also showed the beginnings of consciousness around ‘old age’ stereotypes, such as being old-fashioned and past-it, noted age-based judgement within the work context and, along with the older age groups, also perceived stereotypes about using technology, driving and doing physical activity. Corroborating some of these findings, Study 2 found that middle-aged respondents reported more threat than younger, but not older participants, in the use of technology and alongside older adults, reported greater threat-based concerns about physical activity when compared to the young.

Not all the findings from Study 1 translated into more threat-based concerns among the middle-aged in the broader context of ‘work’. For instance, there was no effect of chronological age group on threat experienced in the workplace, and when using subjective age categorizations, the marginal effect indicated that younger participants reported more threat than middle-aged and older participants. However, the importance of context and stereotype salience was also highlighted by Steele and Aronson (1995). For example, von Hippel et al. (2019) showed that self-reported threat events in the workplace were more commonly reported among ‘young’ (18–30 years old) and ‘older’ workers (50–66 years old) than ‘middle-aged’ workers (31–49 years old) and that perceived threat was problematic for ‘older workers’ alone. However, ‘older workers’ here refers to participants aged 50 and over, whereas our categorizations include these as the ‘middle-aged’ group (32–59 years old). Feeling ‘old’ within the work context may come earlier than in other contexts. More sensitive age-based analyses may be needed in future research dependent on context.

Thus far, the majority of ABST research has examined how the stereotyping of older adults as incompetent affects their performance on memory/cognitive tasks, but also on driving and physical performance (Lamont et al., 2015). In accordance with this, Study 1 showed that early older-aged respondents were the first age group to perceive stereotypes of physical (slow, weak, deaf, etc.), cognitive (less mentally alert, forgetful, senile, etc.) and broader incompetence (e.g. technologically illiterate and bad drivers), which then continued into late older-age. Everyday contexts in which older adult’s perceived age-based judgement were wider ranging than have previously been tested, such as childcare, volunteering, driving and using technology. Study 2 confirmed that older people have greater threat-based concerns than the young in relation to physical activity and technology. It can be concluded that ABST relating to older adults’ competence is important to examine, especially given its potential scope and consequences, and so wider everyday contexts beyond ‘test performance’ should now be examined, such as the use of and engagement with technology and physical activity.

Notably, late older age adults (70+) in Study 1 also reported that they are stereotyped as a burden to society. This was sometimes stated in the broad sense, but often in relation to economic and healthcare resources. This ties in with their belief that others see them as both incompetent but also as ‘well-off’ and therefore an unnecessary burden or drain on society. This kind of stereotype may not be related to typical ABST ‘performance outcomes’ but we propose it may translate into behaviours and actions to avoid being perceived in this way, including avoidance of asking for help or using shared resources and spaces, as suggested by the previous research on disidentification (Davies et al., 2005; von Hippel et al., 2019). Although Study 2 provided no indication that older adults have greater threat-based concerns relating to accessing healthcare or talking to healthcare professionals, it did find that both younger and older participants report more threat-based concern than middle-aged participants in relation to the COVID-19 pandemic. This is likely due to the negative portrayals both age groups have faced during the pandemic, the 70+ as a ‘burden’ due to being ‘at risk’ and in need of protection, and young students as ‘irresponsible’ and a source for the spread of the virus (Ayalon, 2020; Morrow-Howell et al., 2020). For older adults, in particular, who pre-pandemic were already conscious of being seen as a ‘burden’, threat-based concerns may have very real consequences for how they see themselves within society. This shows the very ‘real’ contexts that could be examined within ongoing ABST research.

The findings extend our understanding of ABST by revealing new domains in which threat is experienced and importantly extends the knowledge base to a wider age range of people. This is important given that the perception of age-based judgement and threat-based concern may lead to potentially negative ABST effects on motivation and behaviour that increase inequality experienced between age groups. Advancing our understanding of ABST in this way, beyond artificial test-based settings, is key to understanding its relevance to everyday lives. However, identifying sources of threat-based concern is just the first step. Further research is needed to understand the nature of behavioural and motivational changes that result from threat-based concerns and whether these result in inequalities that can and should be addressed.

## Limitations and Future Research

The average ABST ratings could be considered as low given many are on or below the mid-point of the 1 (strongly disagree) to 7 (strongly agree) scale, potentially indicating that many of the participants were not reporting high levels of threat within some domains. However, it is worth noting that some of the mean levels obtained in this study are reflective of threat concern reported under manipulated ABST conditions. For instance, using two of the same threat concern items participants aged 61 to 95 reported mean levels of 3.46 in Swift et al. (2012a).

Although this study indicates the likelihood that a number of everyday contexts present an age-related threat to young, middle-aged and older adults, it still has yet to be evidenced whether ABST effects occur within these domains. The lack of research in everyday contexts is likely to be due to the difficulty in doing this experimentally. Future research could consider the benefits of qualitative, cross-sectional or longitudinal research methods to expand on ABST within these environments. For instance, research may have to draw inferences from retrospective self-reported data as has been common place in ABST research within the workplace (Kulik et al., 2016; Manzi et al., 2019), a setting in which it would be unethical to elicit ABST experimentally. Other research has used mock situations, such as driving simulators to represent everyday contexts (Lambert et al., 2016).

Exploring ABST outside the laboratory may also require more complex research designs than measuring ABST effects of test-based performance outcomes. As noted, ABST may not just result in poorer performance outcomes but may disadvantage negatively stereotyped age groups if it leads to changes in engagement and motivation, such as keeping-up with modern technology, staying fit, propensity for leadership and asking for help and support (Davies et al., 2005). Capturing this may require novel research designs. Further, everyday stereotype-relevant tasks may not always be as difficult or pressured as the tests typically used within ABST research (Lamont et al., 2015), and automatic or easier tasks may be less affected by changes in emotions, motivations and working memory (Horton et al., 2010; Swift et al., 2012a,b; Chiviacowsky et al., 2018; Marquet et al., 2018; Barber et al., 2020; Chalabaev et al., 2020). It is then a question of whether ABST effects should and can be measured. In favour of measurement, recent evidence has shown that in these contexts, changes in working memory (and by extension other ABST mechanisms) may still occur but, instead of affecting more automated or less difficult outcomes, pose a risk to outcomes beyond the stereotyped task (Chalabaev et al., 2020). It is possible therefore that cognitive depletion caused by ABST may not always affect the act of walking or driving (as examples of everyday stereotyped tasks), but both tasks require simultaneous awareness of a complex environment, such as associated trip and road-related hazards. Some level of heightened emotion and cognitive depletion caused by ABST in these contexts may not affect basic walking/driving, but additional challenges within these contexts may put those experiencing ABST at greater risk of accidents. Again, capturing this within the research design adds additional complexity to examining ABST effects in everyday contexts.

## Conclusion

Previous ABST research has focused on stereotypes of older people as less competent and the impact of this on their cognitive and memory performance (Lamont et al., 2015). The current research extends understanding of perceptions of age-based judgement and threat-based concerns in everyday contexts as precursors to ABST effects. The two studies provide the first exploration of age differences in threat-based concerns, highlighting that ABST research might broaden its scope to look at new age groups, domains and ABST outcomes, including (1) the motivational and behavioural consequences of threat-based concerns for younger adults when driving, in leadership and during the pandemic; (2) cues to ‘old’ age stereotypes and threat-based concerns among late middle-aged adults within the workplace; (3) the role of broad stereotypes of ‘incompetence’ and being ‘past-it’ on middle-aged and older adults’ engagement with technology and physical activity and (4) the potential ABST effects resulting from perceived negative judgement of older people as a burden to society and problem in the context of a national crisis.

## Data Availability Statement

The raw data supporting the conclusions of this article will be made available by the authors, without undue reservation.

## Ethics Statement

The studies involving human participants were reviewed and approved by the University of Kent, School of Psychology Ethics Committee (references: 20122324 and 202016014841796628). The patients/participants provided their written informed consent to participate in this study.

## Author Contributions

RL lead the study development with the input of all co-authors, completed Study 1 data collection and analysis, and wrote the manuscript with the input of all co-authors. HS and LD were second and third coders of Study 1 and completed Study 2 data collection, and HS completed data analysis. All authors contributed to the article and approved the submitted version.

### Conflict of Interest

The authors declare that the research was conducted in the absence of any commercial or financial relationships that could be construed as a potential conflict of interest.
